# Thermodynamic analysis of magnetocaloric properties of ferromagnet undergoing structural phase transition near Curie temperature

**DOI:** 10.1038/s41598-025-15896-8

**Published:** 2025-08-23

**Authors:** Victor A. L’vov, Olga Salyuk, Anna Kosogor

**Affiliations:** 1 V.G. Baryakhtar Institute of Magnetism of the NAS of Ukraine, Kyiv, Ukraine; 2 National Technical University of Ukraine “Igor Sikorsky Kyiv Polytechnic Institute”, Kyiv, Ukraine; 3 Faculty of Physics, University of Vienna, Vienna, Austria; 4 University for Continuing Education Krems, Krems, Austria

**Keywords:** Magnetic energy, Modified Maxwell relation, Magnetization, Heat capacity, Magnetocaloric effect, Temperature change, Materials science, Physics

## Abstract

A thermodynamic analysis of the magnetic, caloric and magnetocaloric properties of the ferromagnets undergoing the structural phase transitions (SPTs) near the Curie temperature has been carried out taking into account the effect of SPT on the energy of magnetic subsystem of ferromagnet. A special emphasis has been placed on the account of minimum conditions for Gibbs free energy and the modification of magnetic Maxwell relations with consideration of the effect of SPT on the magnetic energy. The computations, based on the commonly used equations for the energy and entropy of the magnetic subsystem of ferromagnet, discovered and estimated quantitatively a drastic dependence of magnetic heat capacity and magnetic-field-induced temperature change on the difference between the SPT temperature and Curie point. The performed theoretical analysis is aimed to motivate the systematic experimental research of magnetic and magnetocaloric materials undergoing the SPT at temperatures intentionally tuned close to the Curie point.

## Introduction

Some solid-state compounds and alloys exhibit a magnetocaloric effect (MCE), i.e. a noticeable temperature change caused by application or removal of a magnetic field (see^1^ and references therein). These solids are referred to as magnetocaloric materials. Magnetocaloric materials are considered promising for solid-state refrigeration technologies^1–3^. The purposeful search for materials usable for solid-state refrigeration is an actual task.

Ferromagnets that exhibit the magnetostructural phase transitions form a special type of magnetocaloric materials. The MCE observed in such ferromagnets is conventionally referred to as a “giant MCE”^4^. Consistent experimental research of giant MCE requires significant efforts and expenditures, which can be minimized by preliminary theoretical evaluation of the magnetic-field-induced temperature change observable in such materials. However, such evaluation encounters problems arising during theoretical analysis of magnetostructural transitions (see e.g.^5^).

Two research trends of giant MCE are noticeable among others. First, the research of magnetocaloric properties of quasistoichiometric Heusler alloys, which undergo the structural phase transitions (SPTs) in the temperature range adjusted to Curie temperature ${T_C}$ by the purposeful change of their chemical composition^6–8^. Second, the research of intermetallic rear-earth alloys, which undergo the magnetostructural phase transitions from paramagnetic (PM) to ferromagnetic (FM) state at the temperatures, which are rather close to the boiling point of natural gas, $T \approx 111{\text{ K}}$. (Among them the intermetallics TbAl_2_ (with ${T_C} \approx 105{\text{ K}}$)^9^, TbFeSi^10^, Nd_2_In (both with ${T_C} \approx 110{\text{ K}}$)^11^ and Dy_0.7_Er_0.3_Co_2_ (with ${T_C} \approx 106{\text{ K}}$) can be mentioned^12^. To unite these trends under a common interest in the thermodynamic (particularly magnetocaloric) properties of multiferroic materials, the results of thermodynamic analysis of the magnetic, caloric, and magnetocaloric properties of a representative ferromagnet undergoing a SPT near the Curie temperature are presented below. A special emphasis is placed on:

i) the theoretical analysis of thermodynamic properties of the representative ferromagnet, accounting for the effect of SPTs on the energy of the magnetic subsystem;
ii) the elucidation of the drastic effect of SPTs and external magnetic field on the temperature dependences of magnetization and heat capacity of the equilibrium magnetic subsystem of ferromagnet;
iii) the modification of the commonly known (*orthodox*) magnetic Maxwell relations, to enable their applicability for the quantitative evaluation of the giant MCE in magnetic solids undergoing SPTs;
iv) the computation of magnetic-field-induced temperature change with proper account of the temperature dependences of magnetic energy and heat capacity.

### Thermodynamic equations and basic assumption

The Gibbs free energy of ferromagnet is the sum of internal energy of the magnetic subsystem of this ferromagnet, *U*, the entropy term, $- TS,$ where *T *is temperature, and Zeeman term, $- {\mathbf{MH}}$, where ${\mathbf{H}}$ is external magnetic field, ${\mathbf{M}}$ is magnetization vector. Therefore, Gibbs free energy is expressed as

1 $$G=U - TS - {\mathbf{HM}}.$$

The internal energy of ferromagnet possessing the uniaxial magnetocrystalline anisotropy can be expressed as

2 $$U(T,{\mathbf{M}})= - \frac{1}{2}{J_{ex}}{{\mathbf{y}}^2} - \frac{1}{2}{K_u}y_{z}^{2},$$

where, the first summand is a spin-exchange energy, the second one is a magnetic anisotropy energy, ${J_{ex}}$ is spin-exchange constant, ${K_u}>0$ is the anisotropy constant, ${\mathbf{y}}(T)={\mathbf{M}}(T)/{M_s}$, ${M_s}$ is saturation magnetization, *z*-axis is aligned with the easy magnetization direction.

Let the ferromagnet undergo a SPT above or below the Curie temperature. Generally speaking, the structural changes influence the spin-exchange interaction between magnetic atoms and the magnetic anisotropy. If the magnetic field value exceeds the saturating value ${H_s}$, the magnetization vector is practically parallel to the magnetic field, and therefore, ${y_z}=y\cos \gamma$, where $\gamma =const$ is the angle between the easy magnetization axis of the experimental sample and vector ${\mathbf{H}}$. In this case the internal energy and the Gibbs free energy of the ferromagnet undergoing the SPT are expressed as

3 $$\begin{gathered} U(T,y)= - J(T){y^2}/2, \hfill \\ G(T,y)=U(T,y) - TS(y) - H{M_s}y, \hfill \\ \end{gathered}$$

where

4 $$J(T)={J_m}+\Delta J(T),$$

the constant coefficient ${J_m}={J_{ex}}+{K_u}{\cos ^2}\gamma$ characterises the magnetic (spin-exchange and/or spin-orbit) energy density of the high-temperature phase (see Eq. (2)), the summand $\Delta J(T)$ is introduced in Eq. (4) to take into account the energy change caused by SPT.

The thermodynamic phase corresponding to the minimum of Gibbs potential at $T<{T_{TR}}$ or $T>{T_{TR}}$ is referred to as the low-temperature or high-temperature phase, respectively. Equation (4) expresses the *basic assumption*, which simplifies theoretical analysis of thermodynamic properties of ferromagnet undergoing the SPT. In accordance with this assumption the SPT essentially is an external (with respect to magnetic subsystem) factor, which predetermines the difference between the magnetic energies of the low-temperature and the high-temperature phases. It should be emphasized, first, that the assumption is not true or is at least questionable if the Curie temperature lies in the temperature range of the SPT, because a consistent thermodynamic analysis of magnetostructural phase transitions must be based on the *interrelated* minimum conditions for Gibbs free energy with respect to the magnetic variable *y* and the structural order parameter (elastic strain, electric polarization, etc.); second, the difference between the magnetic energies, $- \Delta J(T){y^2}/2$, may be caused by the impact of structural changes on the spin-exchange energy and/or the magnetic anisotropy energy (see Eq. (4) and definition of parameter ${J_m}$).

The entropy function of the magnetic subsystem of ferromagnet can be expressed as

5 $$S(y)={S_{PM}} - \frac{1}{2}n{k_B}[(1+y)\ln (1+y)+(1 - y)\ln (1 - y)],$$

where ${S_{PM}}$ is the entropy of ferromagnet in paramagnetic state, $n=N/V$, *N* and *V* are the number of magnetic atoms and volume of magnetic solid, respectively, ${k_B}$ is Boltzmann constant. Equation (5) is derived for the statistical ensemble of atoms with spin $s=1/2$ but is widely used for the general description of the magnetic properties of ferromagnets^13,14^.

The temperature dependence of magnetization *in the equilibrium state* of ferromagnet and the appropriate function ${y_{{\text{eql}}}}(T)$ should be found from the minimum principle for Gibbs free energy. The minimum conditions are formulated as

6 $$\partial G/\partial y=0,$$

7 $${\partial ^2}G/\partial {y^2}>0.$$

The Eqs. (5), (6) and the mathematical identity $\ln [(1+y)/(1 - y)]=2\operatorname{ar} \tanh y$ result in the following equation:

8 $$y(T,H)=\tanh \left[ {\frac{{\Theta (T)}}{T}\left( {y+\frac{{{M_s}H}}{{{J_m}+\Delta J(T)}}} \right)} \right],$$

where

9 $$\Theta (T)={T_C}[1+\Delta J(T)/{J_m}].$$

The Eqs. (8), (9) enable the quantitative description of the equilibrium magnetization values ${M_{{\text{eql}}}}(T,H)={y_{{\text{eql}}}}(T,H){M_s}$ inherent to ferromagnets undergoing SPTs in the constant magnetic field (for more details and the relevant examples, see^15,16^). Curie temperature ${T_C}$ is the PM ‒ FM phase transition temperature, corresponding to the high-temperature phase, i.e. the temperature which satisfies the condition ${y_{{\text{eql}}}}({T_C},0)=0$ at $\Delta J=0$; the function $\Theta (T)$ varies from ${T_C}$ to the value $\Theta (0)={T_C}(1+\Delta J(0)/{J_m})$, which is interpreted as the Curie temperature of the low-temperature phase^15,17^.

The heat capacity of ferromagnet can be computed from the Eq. 

10 $${C_P}={C_{{\text{nm}}}}+T{[\partial S({y_{{\text{eql}}}})/\partial T]_{P,H}},$$

where *P* is pressure, ${C_{{\text{nm}}}}$ is non-magnetic part of heat capacity. Outside the temperature range of SPT, the volume *V* can be considered constant due to the smallness of thermal expansion coefficient. Therefore, the approximate equality ${C_P} \approx {C_V}$ and the Eq. 

11 $${C_V}={C_{{\text{nm}}}}+\partial {[U(T,{y_{{\text{eql}}}})/\partial T]_{V,H}},$$

can be used for computations^18^.

Let the magnetic field applied to ferromagnetic solid increase from value ${H_1}$ to value ${H_2}$. If $V(H) \approx V(0)$, a mechanical work performed by the experimental sample due to the magnetic field application can be disregarded, and the first law of thermodynamics results in the approximate equality $T\Delta {S_{12}}(T) \approx \Delta {U_{12}}(T)<<{U_0}$.

### Equations describing the magnetic-field-induced temperature change

Let the magnetic field applied to ferromagnetic solid vary from value ${H_1}$ to value ${H_2}$. If $\delta Q$ denotes the infinitesimal heat evolved/absorbed by unit volume of the solid in the increasing/decreasing magnetic field, the commonly known relationships $\delta Q=TdS$, $\delta Q={C_P}dT$ and $dS=(\partial S/\partial H)dH$ lead to the following equation for the adiabatic temperature change:

12 $$\Delta {T_{1 - 2}}=\int\limits_{{{H_1}}}^{{{H_2}}} {\frac{T}{{{C_P}(T,H)}}\left( {\frac{{\partial S}}{{\partial H}}} \right)} dH.$$

The orthodox Maxwell relation (OMR),

13 $$\partial S/\partial H=\partial M/\partial T$$

is commonly used to replace the entropy with the directly measurable magnetization value, $M(T,H)$. But the basic assumption leads to an explicit dependence of the internal energy on temperature, and therefore, the equations

$$\partial G/\partial T= - S+\partial U/\partial T,$$

14 $$\partial G/\partial H= - M,$$

$${\partial ^2}G/\partial H\partial T={\partial ^2}G/\partial T\partial H,$$

result in the modified Maxwell relation,

15 $$\frac{{\partial S}}{{\partial H}}=\frac{{\partial M}}{{\partial T}}+\frac{{{\partial ^2}U}}{{\partial H\partial T}},$$

and Eqs. 

16 $$\Delta {S_{1 - 2}}=\int\limits_{{{H_1}}}^{{{H_2}}} {\left( {\frac{{\partial M}}{{\partial T}}+\frac{{{\partial ^2}U}}{{\partial H\partial T}}} \right)} dH,$$

17 $$\Delta {T_{1 - 2}}=\int\limits_{{{H_1}}}^{{{H_2}}} {\frac{T}{{{C_P}(T,H)}}\left( {\frac{{\partial M}}{{\partial T}}+\frac{{{\partial ^2}U}}{{\partial H\partial T}}} \right)} dH.$$

If $V(H) \approx V(0)$, the mechanical work performed by the experimental sample due to the magnetic field application can be disregarded, and the first law of thermodynamics results in the approximate Eq. 

18 $$T\Delta {S_{1 - 2}}(T,y) \approx \Delta {U_{1 - 2}}(T,y),$$

which relates the heat absorbed/evolved by the sample to its internal energy. The internal energy density expressed by Eqs. (3, 4) is independent on volume. In this case, Eq. (18) is an exact equality, and the temperature change is expressed as

19 $$\Delta {T_{1 - 2}}=\int\limits_{{{H_1}}}^{{{H_2}}} {\frac{1}{{{C_V}(T,H)}}\left( {\frac{{\partial U}}{{\partial H}}} \right)} dH.$$

Equation (19) may be more convenient for theoretical analysis of MCE, because characteristics of ferromagnet (such as the spin-exchange energy, magnetocrystalline anisotropy energy, the energy of magnetostatic interaction, etc.) can be evaluated theoretically from first principles or estimated from experimental data.

### Impact of structural phase transition on heat capacity and giant magnetocaloric effect

**Fig. 1:**
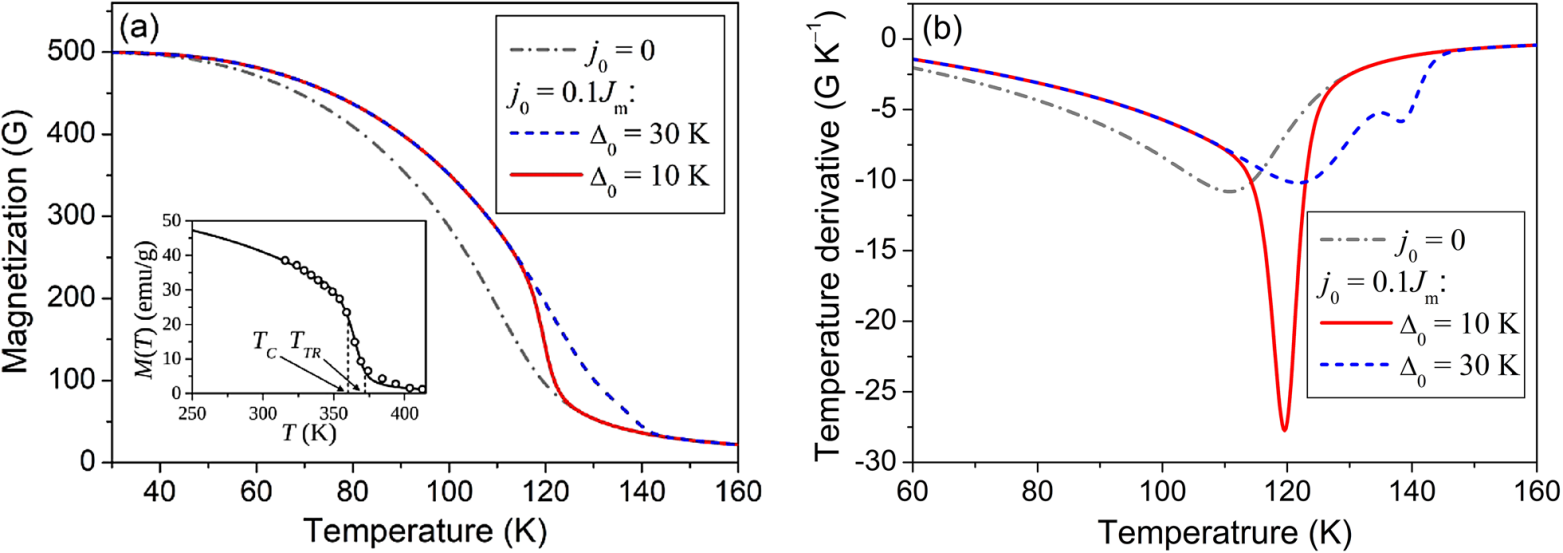
Magnetization functions *M*(*T*) (**a**), and their temperature derivatives *dM*(*T*)/*dT* (**b**), computed using Eq. (8) for ferromagnets undergoing SPTs at 120 K (solid lines) and 140 K (dashed lines), *T*_*C*_ = 110 K. The values computed for the ferromagnet, which does not undergo structural phase transition, are shown by the dash-dotted lines. *Inset*: Temperature dependence of magnetization of Ni_53.1_Mn_26.6_Ga_20.3_ alloy, measured (circles) and computed using Eq. (8) (line).

The SPTs of intermetallics and Heusler alloys typically go through the mixed two-phase states, and therefore, the energy parameter $\Delta J(T)$, which describes the effect of SPT on the magnetic energy, is expressed as

20 $$\Delta J(T)={j_0}\alpha (T),$$

where $\alpha (T)$ is the volume fraction of low-temperature phase, ${j_0}=const$. The volume fraction of the low-temperature phase increases from zero to unit on cooling of ferromagnet and can be modelled by the function

21 $$\alpha (T)=\frac{1}{2}\left[ {1+\tanh \left( {\frac{{{T_{TR}} - T}}{{{\Delta _{TR}}}}} \right)} \right],$$

where the temperature of SPT, ${T_{TR}}$, corresponds to the value $\alpha =1/2$, the parameter ${\Delta _{TR}}$ is the half-width of the temperature range of the mixed two-phase state. The temperature evolution of the volume fractions of phases coexisting in the temperature interval of magnetostructural transitions can be obtained from X-ray powder diffraction measurements and shows a similar dependence to that of the model function (21) (see, e.g^7,19^). The temperatures of SPTs observed in intermetallic compounds and Heusler alloys can be expressed as ${T_{TR}}={T_C}+{\Delta _0}$, where the difference between the temperatures of magnetic and SPTs, ${\Delta _0}$, can be purposefully changed by the variation of chemical composition of the compound/alloy^6–8^. The abrupt change of the magnetization value is commonly recognized as the factor promoting giant MCE. Such change is observed in the case if $|{\Delta _0}/{T_C}|<<1$ (see solid line in Fig. 1). Therefore, this case deserves a detailed consideration.

Figure 1 (a) illustrates the $M(T)$ functions computed from Eqs. (8), (9), (20) and (21); the values used for computations are shown in Table 1; inset illustrates the applicability of Eq. (8) for the quantitative calculation of the temperature dependence of magnetization of real alloys (for the details of the measurements and the calculations see Refs.^17^ and ^16^, respectively).

The ${j_0}/{J_m}$ value shown in Table 1 results in the relationship

22 $$\Theta (T)={T_C}[1+0.1\alpha (T)].$$

The volume fraction of the high-temperature phase decreases from 0.999 to 0.013 on cooling of ferromagnet from the temperature ${T_{TR}}+10\;{\text{K}}$ to the temperature ${T_{TR}} - 10{\text{ K}}$. Therefore, if the SPT temperature satisfies equality $|{T_{TR}} - {T_C}| \geqslant 10{\text{ K}}$, the function $\Theta (T)$ decreases from the value $\Theta ({T_{TR}}+10{\text{ K}}) \approx 120.99{\text{ K}}$ to the value $\Theta ({T_{TR}} - 10{\text{ K}}) \approx 110.01{\text{ K}} \approx {T_C}$. In this case the cooling of ferromagnet induces the SPT from the high-temperature phase in its paramagnetic state to the low-temperature phase in its ferromagnetic state. Due to this, the $M(T)$ function computed for ${\Delta _0}=10\;{\text{K}}$ exhibits a sharp decrease in the temperature range of SPT (see solid line in Fig. 1 (a)). The $M(T)$ function computed for ${\Delta _0}=30\;{\text{K}}$, by contrast, smoothly depends on the temperature, because SPT starts and finishes in the almost pure high-temperature state with Curie temperature ${\tilde {T}_C} \approx 121{\text{ K}}$ (see dashed line in Fig. 1 (a)). The dash-dotted line in Fig. 1 (a) presents the magnetization function computed the ferromagnet with ${T_C}=110{\text{ K}}$, which does not exhibit SPT.

**Table 1:** Parameters used for computation of the temperature dependences of magnetization.

M_S_(0) (G)	T_C_ (K)	J_m_ (erg/cm^3)^	j_0_/J_m_	Δ_TR_ (K)	H (kOe)
500	110	5⋅10^8*^	0.1	3^**^	20^***^

* Corresponds to the Weiss field *J*_*m*_/*M*_*S*_ (0) = 10^3^ kOe, typical for ferromagnets with Curie temperature of about 100 K.

** Typical for the SPTs observed in intermetallic rear-earth alloys and shape memory alloys.

*** Achievable in the experiments with permanent magnets.

It should be mentioned, first, that the minimum point of the temperature derivative of $M(T)$ function is traditionally used for evaluation of Curie temperature. Figure 1 (b) obviously shows that such evaluation is approximately valid for the ferromagnet which does not exhibit SPT (see dash-dotted line in Fig. 1 (b)). If the ferromagnet undergoes SPT, the derivative of $M(T)$ has minimum near the SPT temperature (see solid line in Fig. 1 (b)), or two minimums, first, near the ${T_{TR}}$, and second, between ${T_{TR}}$ and ${T_C}$ (see dashed line).

It should be stressed, second, that according to the phase transition theory the external magnetic field “abolishes” a second-order phase transition from ferromagnetic to paramagnetic phase, because the magnetization value is not equal to zero above the Curie temperature. Therefore, the determination of the magnetic-field-induced shift of Curie temperature is either meaningless procedure or the determination of magnetic-field-induced shift of the SPT temperature.

**Fig. 2:**
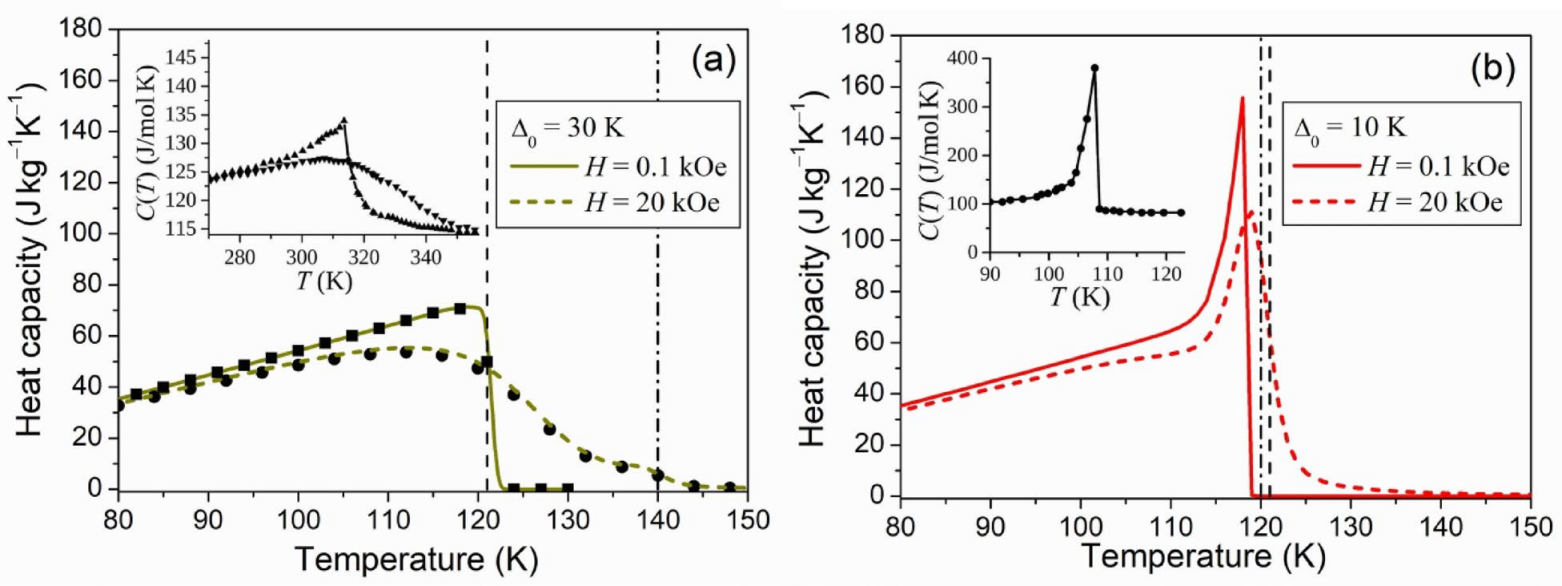
Computed temperature dependencies of the heat capacities, *C*_*P*_ (lines), *C*_*V*_ (symbols), of the magnetic subsystem of ferromagnets, undergoing the temperature-induced structural transitions in the stationary magnetic field. Vertical dashed and dash-dotted lines mark the values *T*_*C*_ and *T*_*TR*_, respectively. *Insets*: Temperature dependences of the heat capacity of Ni_2_Mn_1.4_Sn_0.6_ measured in the magnetic fields of 0 kOe and 20 kOe^24^(**a**); the heat capacity of Nd_2_In^11^(**b**).

A substitution of computed magnetization values in Eqs. (10), (11) enables the computation of temperature dependence of heat capacity observed in the equilibrium thermodynamic processes. Figure 2 shows the contribution of magnetic subsystem of ferromagnet to the heat capacities ${C_P}(T)$ and ${C_V}(T)$, computed from Eqs. (10), (11), respectively. The insets illustrate the resemblance between the typical temperature dependencies of heat capacities, observed in the experiments, and the theoretical ones. The Eqs. (10), (11) yielded the same results, because the Gibbs free energy expressed by Eqs. (1), (2) does not involve elastic energy, and therefore, the mechanical work performed by elastic forces is disregarded. Figure 2 (a) shows the orthodox temperature dependencies of heat capacity, reported elsewhere (see e.g.^20^). The only feature caused by the SPT is a faintly visible plateau in the graph plotted for the external magnetic field of 20 kOe. In contrast, Fig. 2 (b) shows the sharp peak of heat capacity in the temperature range of SPT. When the SPT temperature appeared to be close to Curie temperature of the high-temperature phase, theoretical curves clearly reflect the peculiarities of experimental ones obtained in Refs.^21–23^ for the ferromagnets exhibiting magnetostructural phase transitions.

In the external field of 0.1 kOe the heat capacity shown in Fig. 2 (a) by the solid line decreases almost to zero at the temperature ${\tilde {T}_C}=121 \pm 0.5{\text{ K}}$, i.e., at the Curie temperature of low-temperature phase. The heat capacity shown by the solid line in Fig. 2 (b) reaches maximum at the temperature of $118 \pm 0.5{\text{ K}}$. The temperature derivative of magnetization shown by the solid line in Fig. 1 (b) reaches maximum at the temperature of $119.5 \pm 0.5{\text{ K}}$. All temperatures mentioned here are noticeably higher than the Curie temperature of high-temperature phase, that is the temperature ${T_C}$, involved into Eq. (9).

**Fig. 3:**
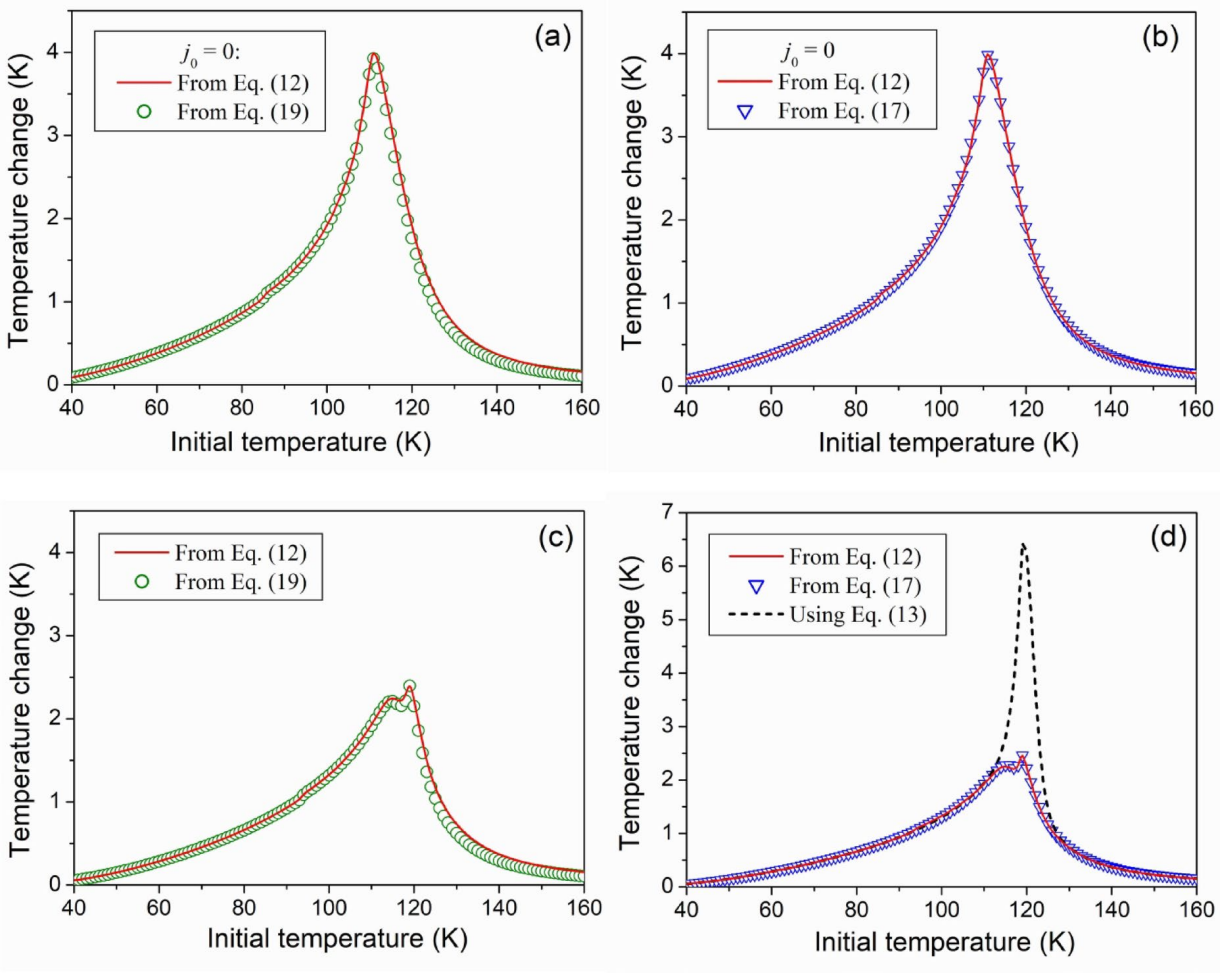
Adiabatic temperature change caused by the magnetic field change from 100 Oe to 20 kOe, computed for the ferromagnet, which does not undergo structural transition, (**a**), (**b**), and ferromagnet undergoing structural phase transition at *T*_*TR*_ = 120 K (**c**), (**d**).

A substitution of the computed values of heat capacity into Eqs. (12), (17) or (19) enables three different ways of calculating the magnetic-field-induced temperature change. Figure 3 shows that these ways lead to the same result, which is strongly different from the result obtained using the OMR, Eq. (13). Therefore, this figure obviously demonstrates an internal consistency of the equation system (2)–(5), (8), (9), which describes the magnetic properties of ferromagnet, with commonly known thermodynamic relationships and *modified* Maxwell relation Eq. (15). Moreover, comparing the graphs shown in Figs. 3 (a), (b) with those presented in Figs. 3 (c), (d), one can see that the theory discovers a *possibility of suppression* of MCE by SPT, if this SPT results in the increase of the internal energy of magnetic subsystem of ferromagnet in the low-temperature phase. It should be stressed, that such SPTs are observed in the widely studied ferromagnetic shape memory alloys (see^17^ and review article^16^).

**Fig. 4:**
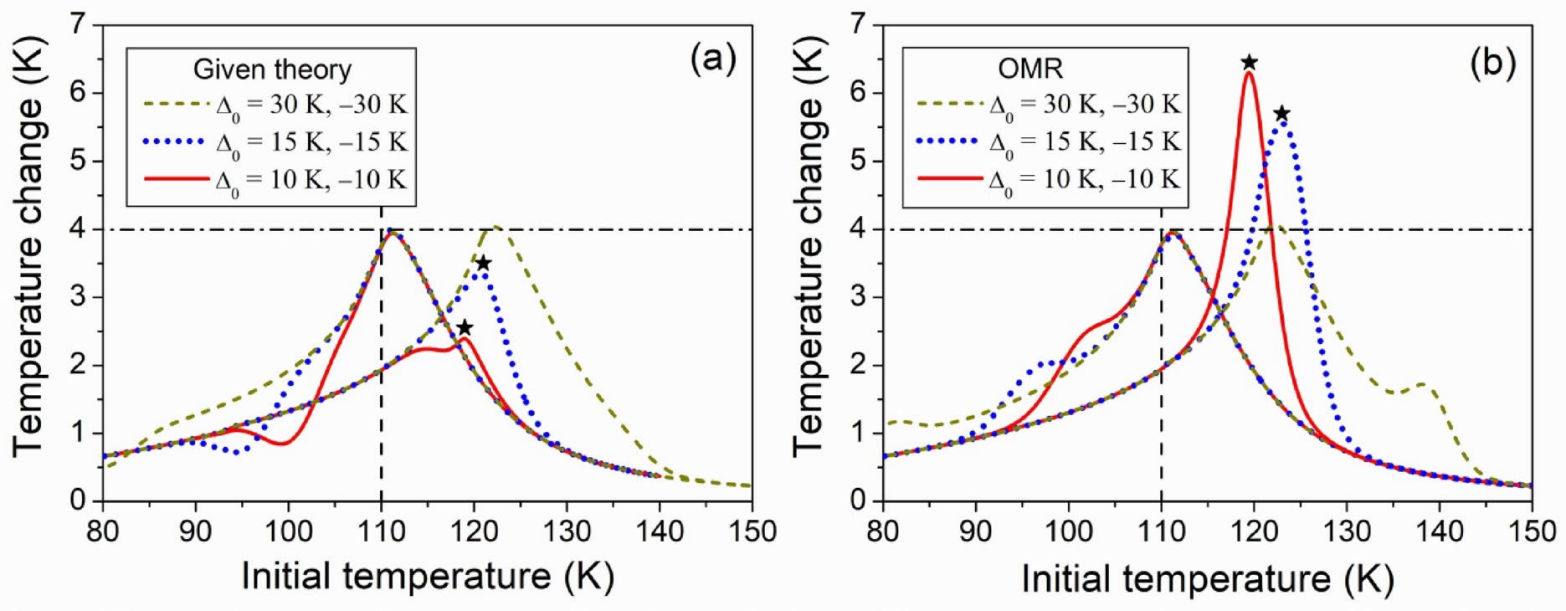
Adiabatic temperature change caused by the magnetic field change from 100 Oe to 20 kOe as a function of initial temperature of ferromagnet; the computations were carried out using Eq. (12) (panel (**a**)), and orthodox Maxwell relations (panel (**b**)). The curves with maxima near *T*_*C*_ = 110 K correspond to negative values Δ_0_, the curves with maxima near 120, 125, and 140 K correspond to positive Δ_0_ values. The horizontal dash-dotted line shows the maximum temperature change computed for the alloy, which does not undergo the structural transition (i.e., for *j*_0_ = 0), the vertical dashed line shows the Curie temperature, the stars indicate the points discovering the maximum difference between the results of given theory and orthodox one.

The coupling between magnetic and structural transitions is considered a key strategy for achieving a giant MCE. Such coupling has been successfully realized in many magnetic systems through chemical substitutions^6–8^. Therefore, it is of practical interest to theoretically analyze how the adiabatic temperature change depends on the temperature interval ${\Delta _0}={T_{TR}} - {T_C}$, which is the difference between the Curie point and SPT temperature. To this end, the $\Delta {T_{1 - 2}}(T)$ functions were computed for different values of ${\Delta _0}$. Figure 4 shows the results obtained for the representative ferromagnet described above. As far as Eqs. (12), (17) and (19) lead to the same results, these results are referred to as the results of given theory.

Figure 4 (a) shows the results of computations carried out in the framework of given theory. Comparing the lines presented in the same format and colour one can see the drastic dependence of MCE on the sign of ${T_{TR}} - {T_C}$ value. It is seen, moreover, that the maximum values of $\Delta {T_{1 - 2}}(T)$ computed for the ferromagnets undergoing the SPTs do not exceed the maximum value of 4.0 K computed for ${j_0}=0$, i.e., for the ferromagnet, which does not undergo SPT. If ${T_{TR}}<{T_C}$ the SPT does not noticeable influence the maximum value of $\Delta {T_{1 - 2}}(T)$, but noticeably lowers this value in the opposite case (by factors of about 1.6 or 1.2, if ${\Delta _0}$ is equal to 10–15 K, respectively). The maximum value computed for ${\Delta _0}=30\;{\text{K}}$ is close to 4.0 K, because SPT occurs far from PM – FM phase transition.

**Fig. 5:**
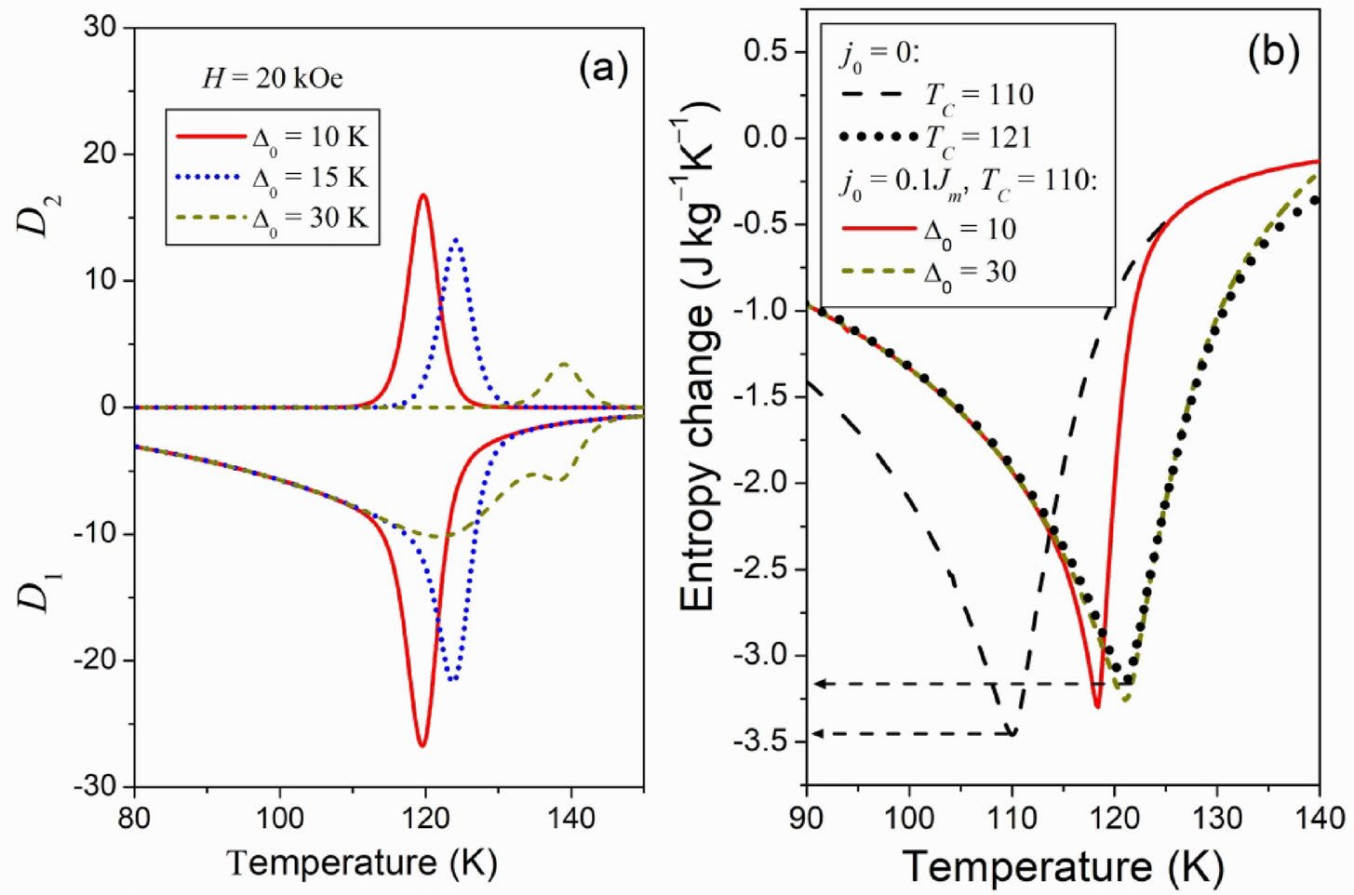
Derivatives *D*_1_ and *D*_2_ included in the modified Maxwell relation (Eq.(17)), computed for various Δ_0_ values (**a**). Magnetic-field-induced entropy change computed for different *T*_*C*_ and Δ_0_ values (**b**).

Figure 4 (b) shows the results of computations carried out using OMR. This figure shows that the $\Delta {T_H}(T)$ graphs resulting from OMR demonstrate resemblance to those shown in Fig. 4 (a) if ${\Delta _0}=30\;{\text{K}}$. However, the use of OMR leads to considerable overestimation of $\Delta {T_{1 - 2}}(T)$ resulting from the given theory if ${\Delta _0} \leqslant 15$. A disagreement of two ways of estimation of MCE can be explained formally with help of modified Maxwell relation. This relation was used to obtain Eq. (17), which expresses the temperature change through the sum of derivatives ${D_1}(T)=\partial M/\partial T$ and ${D_2}={\partial ^2}U/\partial H\partial T$. The derivatives have different signs (see Fig. 5 (a)) and therefore $|{D_1}+{D_2}|<|{D_1}|$. In the equation resulting from OMR, the derivative ${D_2}$ is absent, and due to this, the use of OMR leads to the overestimation of the temperature change.

As mentioned above, the magnetic-field-induced entropy change is considered the main characteristic of MCE. This characteristic is shown in Fig. 5 (b). It is seen that the SPT reduces the entropy change if the temperature of SPT is close to Curie temperature of the high-temperature phase, ${T_C}=110\;{\text{K}}$. (Compare the curve plotted for ${\Delta _0}=10{\text{ K}}$ with the curve corresponding to ${j_0}=0$). If the temperature of SPT is much higher than ${T_C}$, the entropy change is approximately equal to the entropy change computed for the high temperature phase, that is for the ferromagnet with ${\tilde {T}_C}=121{\text{ K}}$. (Compare the curve plotted for ${T_C}$, ${\Delta _0}=30{\text{ K}}$ with the curve corresponding to ${\tilde {T}_C}$, ${j_0}=0$). The horizontal arrows show that the relative difference between the maximum absolute values of the entropy changes computed for two phases is close to 0.09. This value is close to the relative difference of Curie temperatures $({\tilde {T}_C} - {T_C})/{\tilde {T}_C} \approx 0.091$. The computations carried out for ${T_C}=110{\text{ K}}$, 100 K and 80 K, without changing the other parameters, show that in the absence of magnetostructural phase transitions, the increase of the maximum absolute value of the entropy change is strictly proportional to the lowering of Curie temperature. This *preliminary* conclusion deserves a special theoretical analysis and experimental verification.

## Summary and discussion

Commonly known magnetic Maxwell relations, called above the orthodox Maxwell relations, are widely used for evaluation of giant magnetocaloric effect. It is important, however, that OMR are derived for thermodynamic processes, which go through the subsequent equilibrium states of thermodynamic system, while the giant MCE is the MCE preconditioned by magnetostructural phase transition. The evaluation of magnetic-field-induced temperature change on the base of OMR ignores the phase transition process between two equilibrium thermodynamic states. As far as the general self-consistent theory of magnetostructural phase transitions is not created until now, the thermodynamic properties of “representative ferromagnet” whose Curie point lies outside the temperature range of SPT have been analyzed and the modified Maxwell relations were derived, assuming that the SPT results in the explicit temperature dependence of the energy density of the magnetic subsystem of ferromagnet. The *given approach* to theoretical analysis of magnetic and magnetocaloric properties of ferromagnets is based on this assumption and on the fundamental thermodynamic relationships. This analysis resulted in:

i) the theoretical temperature dependencies of magnetization (Fig. 1), which are in agreement with dependencies inherent to the real ferromagnets undergoing the SPTs (see inset in Fig. 1 and Ref.^16^);
ii) the theoretical curves of heat capacity ${C_P}(T)$ (Fig. 2), which reproduce the sharp narrow peaks on the experimental curves taken for the ferromagnets undergoing SPTs on cooling in the constant magnetic field (see insets in Fig. 2 and Ref.^11^);
iii) theoretical values of the magnetic-field-induced temperature change $\Delta {T_H}$ computed for the different intervals ${T_{TR}} - {T_C}$ between the temperature of SPT and Curie temperature.

It has been stressed, that computed $\Delta {T_H}$ values are rather close to those resulting from the OMR, if the temperature interval ${T_{TR}} - {T_C}$ is much wider than the temperature interval of SPT. However, a drastic contradiction between the results of given theory and the theory based on OMR arises if ${T_{TR}}$ approaches ${T_C}$: the given theory predicts the suppression of MCE by the SPT that increases the energy of magnetic subsystem of ferromagnet, while the orthodox theory predicts the enhancement of MCE.

It should be noted that the given approach to the theoretical analysis of MCE is promising for the elucidation of the interrelation between MCE and the basic properties of ferromagnets exhibiting SPT. The properties, related to the Curie temperature through the spin-exchange process and magnetic anisotropy (large magnetostriction, spontaneous or forced straining of crystal lattice, the displacements of the magnetic atoms inside the unit cells of the crystal lattice, etc.) can be taken into account. These properties can be related to multicaloric effects^25^.

It should be emphasised that the inverse MCE related to the SPTs of ferroelastic type in the metamagnetic alloys is widely studied^26–28^. It is argued that the spontaneous deformation of the crystal lattice weakens the inverse MCE observed in these alloys^29^. The results obtained in this article suggest the possibility of similar magnetocaloric property of the materials exhibiting normal magnetocaloric effect. However, the application of given theoretical approach to ferromagnets exhibiting the phase transitions from high-temperature ferromagnetic to the low-temperature paramagnetic^27,30^, ferrimagnetic^31^ or antiferromagnetic^32–34^ phases needs the detailed experimental data about the magnetic structure of the high-temperature and low-temperature states of ferromagnets with the competing spin-exchange interactions.

Finally, the magnetic solids exhibiting giant MCE at the liquid helium and sub-liquid helium temperatures worth mentioning (see Refs.^35–37^ and references therein). Such solids are promising for the solid-state helium temperature refrigeration. An applicability of the given theoretical approach to the description of MCE in such solids is questionable and needs special consideration.

## Conclusion

A thermodynamic analysis based on the commonly used equations for the energy and entropy of the magnetic subsystem of ferromagnet showed how the structural phase transition occurring near the Curie point impacts the magnetic, caloric and magnetocaloric properties of the ferromagnet. The analysis discovered a sharp dependence of these properties on the difference between the SPT temperature and Curie point. The obtained results can motivate the systematic experimental research of magnetic and magnetocaloric materials undergoing the SPT at the temperature, which can be purposefully approached to Curie point (see e.g., the experiments presented in Refs.^6–8^).

## Data Availability

The datasets used and/or analyzed during the current study available from the corresponding author and Victor L’vov on reasonable request.

## References

[1] Franco, V. et al. Magnetocaloric effect: from materials research to refrigeration devices. *Prog Mater. Sci.* **93**, 112–232. 10.1016/j.pmatsci.2017.10.005 (2018).

[2] Gutfleisch, O. et al. Magnetic materials and devices for the 21st century: stronger, lighter, and more energy efficient. *Adv. Mater.* **23**, 821–842. 10.1002/adma.201002180 (2011). 21294168 10.1002/adma.201002180

[3] Gottschall, T. et al. Making a cool choice: the materials library of magnetic refrigeration. *Adv. Energy Mater.* **9**, 1901322. 10.1002/aenm.201901322 (2019).

[4] Chernenko, V. A., L’vov, V. A., Cesari, E. & Barandiaran, J. M. Fundamentals of magnetocaloric effect in magnetic shape memory alloys. in *Handbook Magn. Materials* **28** 1–45 (Elsevier, 2019). 10.1016/bs.hmm.2019.03.001

[5] Kato, T., Nagai, K. & Aisaka, T. A model of magneto-structural phase transition in MnAs. *J. Phys. C Solid State Phys.* **16**, 3183. 10.1088/0022-3719/16/16/020 (1983).

[6] Liu, J. et al. Realization of magnetostructural coupling by modifying structural transitions in MnNiSi-CoNiGe system with a wide Curie-temperature window. *Sci. Rep.* 6, 23386 (2016). 10.1038/srep23386 (2016). 10.1038/srep23386 PMC4793218 26979284

[7] Biswas, A. et al. Designed materials with the giant magnetocaloric effect near room temperature. *Acta Mater.* **180**, 341–348. 10.1016/j.actamat.2019.09.023 (2019).

[8] Pal, S. K. et al. Enhancing giant magnetocaloric effect near room temperature by inducing magnetostructural coupling in Cu-doped MnCoGe. *Mater. Des.* **195**, 109036. 10.1016/j.matdes.2020.109036 (2020).

[9] Wang, F. W., Zhang, X. X. & Hu, F. X. Large magnetic entropy change in TbAl_2_ and (Tb_0.4_Gd_0.6_)Al_2_. *Appl. Phys. Lett.* **77**, 1360–1362. 10.1063/1.1290389 (2000).

[10] Zhang, H. et al. Large magnetocaloric effects of RFeSi (R = Tb and Dy) compounds for magnetic refrigeration in nitrogen and natural gas liquefaction. *Appl. Phys. Lett.* **103**, 202412. 10.1063/1.4832218 (2013).

[11] Biswas, A. et al. Unusual first-order magnetic phase transition and large magnetocaloric effect in Nd_2_In. *Phys. Rev. Mater.* **6**, 114406. 10.1103/PhysRevMaterials.6.114406 (2022).

[12] Ćwik, J., Palewski, T., Nenkov, K., Gutfleisch, O. & Klamut, J. The influence of Er substitution on magnetic and magnetocaloric properties of Dy_1 – x_Er_x_Co_2_ solid solutions. *Intermetallics* **19**, 1656–1660. 10.1016/j.intermet.2011.07.012 (2011).

[13] Isihara, A. *Statistical Physics* (Academic, 2013).

[14] Ziman, J. M. *Principles of the Theory of Solids* (Cambridge University Press, 1972).

[15] L’vov, V. A. & Salyuk, O. Thermodynamic model of quasi-first-order paramagnetic–ferromagnetic phase transition and giant magnetocaloric effect. *Low Temp. Phys.* **51**, 300–305. 10.1063/10.0035811 (2025).

[16] L’vov, V. Effects of magnetoelastic coupling in ferromagnetic shape memory alloys and piezopolymer/fsma composite material. *Low. Temp. Phys.* **51**, 1030–1039 10.1063/10.0037097 (2025).

[17] Chernenko, V., L’vov, V., Zagorodnyuk, S. & Takagi, T. Ferromagnetism of thermoelastic martensites: theory and experiment. *Phys. Rev. B*. **67**, 064407. 10.1103/PhysRevB.67.064407 (2003).

[18] Landau, L. D. & Lifshitz, E. M. *Course of Theoretical Physics* (Elsevier, 2013).

[19] Ghorai, S. et al. Giant magnetocaloric effect in the (Mn,Fe)NiSi-system. *Phys. Rev. Mater.* **8**, 124401. 10.1103/PhysRevMaterials.8.124401 (2024).

[20] Dan’kov, S. Y., Tishin, A. M., Pecharsky, V. K. & Gschneidner, K. A. Magnetic phase transitions and the magnetothermal properties of gadolinium. *Phys. Rev. B*. **57**, 3478–3490. 10.1103/PhysRevB.57.3478 (1998).

[21] Pecharsky, V. K. & Gschneidner, K. A. Giant magnetocaloric effect in Gd_5_Si_2_Ge_2_. *Phys. Rev. Lett.* **78**10.1103/PhysRevLett.78.4494 (1997).

[22] Gràcia-Condal, A. et al. Magnetic and structural entropy contributions to the multicaloric effects in Ni-Mn-Ga-Cu. *Phys. Rev. Mater.* **6**, 084403. 10.1103/PhysRevMaterials.6.084403 (2022).

[23] Tang, X. et al. Magnetic refrigeration material operating at a full temperature range required for hydrogen liquefaction. *Nat. Commun.* **13**, 1817. 10.1038/s41467-022-29340-2 (2022). 35361763 10.1038/s41467-022-29340-2 PMC8971455

[24] Kosogor, A., Barandiaran, J. M., L’vov, V. A., Fernandez, J. R. & Chernenko, V. A. Magnetic and nonmagnetic contributions to the heat capacity of metamagnetic shape memory alloy. *J. Appl. Phys.* **121**, 183901. 10.1063/1.4983025 (2017).

[25] Stern-Taulats, E. et al. Multicaloric materials and effects. *MRS Bull.* **43**, 295–299. 10.1557/mrs.2018.72 (2018).

[26] Krenke, T. et al. Inverse magnetocaloric effect in ferromagnetic Ni–Mn–Sn alloys. *Nat. Mater.* **4**, 450–454. 10.1038/nmat1395 (2005). 15895096 10.1038/nmat1395

[27] Kihara, T., Xu, X., Ito, W., Kainuma, R. & Tokunaga, M. Direct measurements of inverse magnetocaloric effects in metamagnetic shape-memory alloy NiCoMnIn. *Phys. Rev. B*. **90**, 214409. 10.1103/PhysRevB.90.214409 (2014).

[28] Mejía, C. S. et al. On the high-field characterization of magnetocaloric materials using pulsed magnetic fields. *J. Phys. Energy*. **5**, 034006. 10.1088/2515–7655/acd47d (2023).

[29] Gottschall, T., Skokov, K. P., Benke, D., Gruner, M. E. & Gutfleisch, O. Contradictory role of the magnetic contribution in inverse magnetocaloric heusler materials. *Phys. Rev. B*. **93**, 184431. 10.1103/PhysRevB.93.184431 (2016).

[30] Sannigrahi, J. et al. Magnetic States of Ni-Mn-Sn based shape memory alloy: A combined Muon spin relaxation and neutron diffraction study. *Phys. Rev. B*. **99**, 224401. 10.1103/PhysRevB.99.224401 (2019).

[31] Semboshi, S., Umetsu, R. Y., Kawahito, Y. & Akai, H. A new type of half-metallic fully compensated ferrimagnet. *Sci. Rep.* **12**, 10687. 10.1038/s41598-022-14561-8 (2022). 35739287 10.1038/s41598-022-14561-8 PMC9226010

[32] Nikitin, S. et al. The magnetocaloric effect in Fe_49_Rh_51_ compound. *Phys. Lett. A*. **148**, 363–366. 10.1016/0375-9601(90)90819-A (1990).

[33] Aksoy, S., Acet, M., Deen, P., Mañosa, L. & Planes, A. Magnetic correlations in martensitic Ni-Mn-based heusler shape-memory alloys: neutron polarization analysis. *Phys. Rev. B—Condensed Matter Mater. Phys.* **79**, 212401. 10.1103/PhysRevB.79.212401 (2009).

[34] Kosogor, A., Umetsu, R. Y., Golub, V., Xu, X. & Kainuma, R. Magnetic properties, phase diagram and low-temperature specific heat of Ni_50_Mn_50-x_Sb_x_ alloys. *J. Alloys Compd.* **988**, 174130. 10.1016/j.jallcom.2024.174130 (2024).

[35] Zhang, Y., Na, Y., Xie, Y. & Zhao, X. Unveiling the structural and magnetic properties of RENaGeO_4_ (RE = Gd, dy, and Ho) oxides and remarkable low-temperature magnetocaloric responses in GdNaGeO_4_ oxide. *J. Mater. Chem. A*. 10.1039/D5TA00892A (2025).

[36] Zhang, Y., Li, A., Hao, W., Li, H. F. & Li, L. Apatite-type gadolinium-based dense MGd_4_Si_3_O_13_ (M = Mg, ca, and Sr) ceramics: an emerging class of sub-liquid helium temperature magnetic refrigerant. *Acta Mater.* **292**, 121033. 10.1016/j.actamat.2025.121033 (2025).

[37] Chen, F., Xu, J., Zhao, X., Na, Y. & Zhang, Y. Structural and magnetic characterization of weberite-type RE_3_NbO_7_ (RE = Gd, dy, ho, and Er) ceramics with notable cryogenic magnetocaloric responses. *Sci. China Mater.* 1–13. 10.1007/s40843-025-3468-4 (2025).

